# Racial Differences in Utilization of and Factors Contributing to the Use of Knee Replacement Surgery as a Treatment for Severe Knee Osteoarthritis: A Scoping Review

**DOI:** 10.1016/j.ocarto.2026.100844

**Published:** 2026-06-22

**Authors:** Samuel D. Good, Max Krall, Collin Brantner, Diyu Pearce-Fisher, Cynthia Kahlenberg, Ajay Premkumar, Michelle Demetres, Susan Goodman, John D. FitzGerald

**Affiliations:** a David Geffen School of Medicine, University of California Los Angeles, Los Angeles, CA, USA; b Hospital for Special Surgery, New York, NY, USA; c Weill Cornell Medicine, New York, NY, USA; d Department of Orthopaedic Surgery, Emory University School of Medicine, Atlanta, GA, USA; e Stony Brook University, Stony Brook, NY, USA; f Veteran Affairs, Greater Los Angeles Healthcare System, Los Angeles, CA, USA

## Abstract

**Objective:**

Differences in utilization of total knee arthroplasty (TKA) for Black patients with severe knee osteoarthritis (OA) have been well described. Understanding the factors that contribute to these differences is important when considering interventions to optimize TKA utilization. To evaluate these features, we developed a conceptual model for factors influencing TKA utilization and conducted a scoping literature review.

**Methods:**

Literature searches were conducted in April 2019 and April 2020 then updated in October 2022 and January 2024. Our primary outcomes were utilization of TKA for treatment of severe knee OA and factors affecting provider and patient decision making around TKA. We provide qualitative summaries of articles derived from our conceptual model.

**Results:**

Black patients have lower utilization of TKA for treatment of their severe knee OA than White patients with knee OA and worse pain measures at the time of surgery. Large secondary datasets demonstrate that Black patients have increased rates of revision surgery, higher rates of non-home discharges, higher readmission rates and higher episode of care cost. As reported in qualitative studies, Black patients have less favorable perceptions of the benefits of TKA and a lower degree of trust in the healthcare system. Recent policies to reduce cost and/or improve quality of care may have unintended impact on access to care for Black patients.

**Conclusion:**

There are racial disparities between Black and White patients at multiple steps in the decision-making process for TKA. Efforts to reduce these disparities will need to target multiple points contributing to this differential care.

## 1 Introduction

Total knee arthroplasty (TKA) is a highly effective treatment for refractory knee osteoarthritis (OA), with significant improvements in pain and functional outcomes post-operatively [1,2]. However, there are well documented racial disparities in rates of TKA, with Black patients undergoing TKA for knee OA at significantly lower rates when compared with White patients. This was first demonstrated by Skinner et al., in 2003, with multiple successive investigations confirming these results in different populations [[3], [4], [5], [6]].

**Fig. 1:**
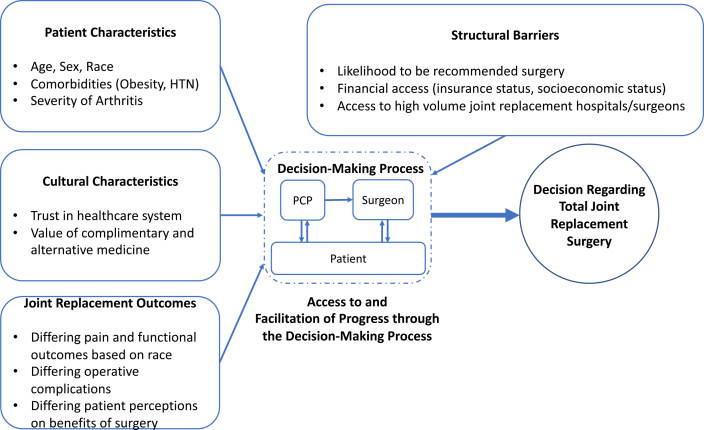
Conceptual model for evaluating disparities in knee replacement.

To better understand the disparity in TKA utilization and the factors contributing to that utilization, we performed a scoping literature review and derived a conceptual model to address the factors that contribute to the patient-provider decision-making process (Fig. 1). These factors include patient characteristics, cultural characteristics, and structural barriers. Quality of the shared decision-making process, including access to high quality providers and hospitals and any biases in recommending surgery are also considered.

## 2 Methods

We followed the Preferred Reporting Items for Systematic Reviews and Meta-Analyses extension for Scoping Reviews (PRISMA-ScR; checklist can be found in Supplement 2) and registered this study with the International Prospective Register of Systematic Reviews with ID number CRD42020132550. We utilized Covidence, an online literature review management platform, to assist with study selection. This study was initially planned as a systematic literature review but evolved over time into a scoping review.

**Fig. 2:**
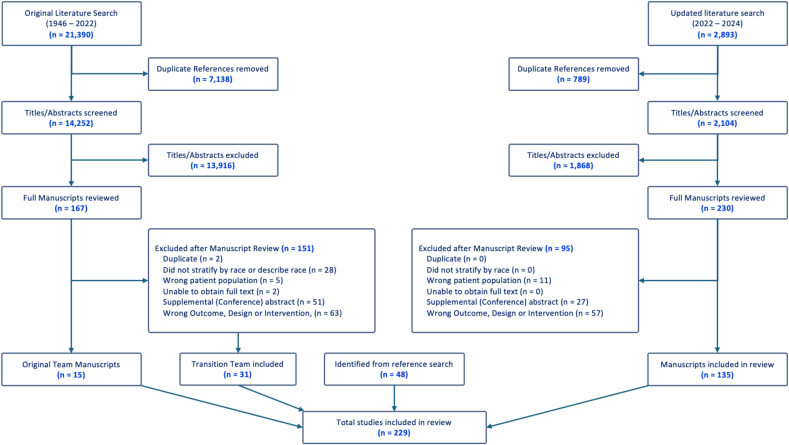
PRISMA diagram from inception and transition team literature searches.

A medical librarian performed comprehensive searches to identify articles (Fig. 2). The literature reviews were conducted in two phases. Searches by the inception team were conducted on April 8, 2019 and, April 7, 2020. The transition team updated searches with the same medical librarian on October 11, 2022 and January 18, 2024 in the following databases: Ovid MEDLINE (1946 to January 2024); Ovid EMBASE (1974 to January 2024); and The Cochrane Library (Wiley). The search strategy included all appropriate controlled vocabulary and keywords for the concepts of “knee replacement arthroplasty,” “osteoarthritis,” “African Americans,” “patient preferences,” and “race.” The search was limited to English language studies (full criteria in Prospective Register of Systematic Reviews registry). To limit publication bias, there were no publication date or article type restrictions on the search strategy.

From the inception team, 3 reviewers (CB, CK, and AP) screened titles and abstracts against pre-defined inclusion/exclusion criteria, and selected articles for full text review. For the transition team, 2 reviewers (MK and SDG) screened the new titles and abstracts, and to ensure a uniform abstraction methodology rescreened titles for full text review and updated inclusion. Case reports, case series, conference abstracts, and studies with patient cohorts exclusively outside of the United States were excluded from the review. Any discrepancies between reviewers’ inclusion decisions were resolved by a third reviewer (SG for inception and JF for transition team).

Full manuscripts were reviewed for all titles and abstracts meeting inclusion criteria. Manuscripts that subsequently did not match inclusion criteria or contained insufficient data were subsequently excluded. Despite identical screening strategies, there were differences in inclusion rates between the teams. With the larger number of years, results from the inception team included a much larger volume of titles than the transition team updated search. The transition team had higher inclusion rates at both title & abstract screenings (11% vs. 1.2%) as well as manuscript screenings (59% vs. 10%) compared to the inception team. To reduce differential selection bias between the teams, the transition team accepted the results of the inception title-abstract screening but then re-screened the 151 manuscripts initially rejected by the inception team.

There was significant heterogeneity across the descriptions of disparities and contributing factors. Therefore, we elected to provide qualitative summaries by domain rather than attempt meta-analyses. Furthermore, outcome measures utilized across studies, including pain, functional impairment, and radiographic evaluation were disparate, making direct comparison impossible. The findings reported here do not suggest uniformity of experience or behavior among Black patients. We recognize that racial identity encompasses substantial within-group diversity, and results should not be interpreted as generalizations about any individual or subgroup.

## 3 Results

Searches across the chosen databases retrieved 14,252 references (1946–2022) and 2104 (2022–2024) after de-duplication. The original team selected 167 manuscripts for review (1.2% inclusion rate for full manuscript review) and 16 manuscripts in their final set. The transition team identified 230 manuscripts for review from their title/abstract list (11% inclusion rate) resulting in 135 manuscripts for inclusion, plus an additional 31 manuscripts included (from the 151 manuscripts initially rejected by the inception team initially team) and an additional 48 manuscripts identified from reference searches for a total of 229 manuscripts in this combined review (see Fig. 2). These manuscripts were assigned to one of 5 major domains (as modeled in Fig. 1): Knee Replacement Surgery Utilization (our primary outcome of interest), Knee Replacement Outcomes, Patient Characteristics, Cultural Characteristics, and Structural Barriers to TKA.

### 3.1 Total Knee Replacement Surgery utilization

Our primary outcome of interest was differences in TKA utilization rates between Black and White patients. The landmark 2003 study by Skinner and colleagues demonstrated that among a national sample of 403,251 Medicare beneficiaries, TKA rates for Black males were less than half that of non-Hispanic White males [6]. This study was replicated in the Veterans Administration (national dataset) in 260,856 OA patients with the benefit of comorbidity data available [7]. The utilization of TKA for Black vs. White Veterans after adjusting for comorbidity remained low (OR = 0.72, 95% CI 0.65–0.80). A recent meta-analysis confirmed that disparities persist today [8].

Disparities in utilization have persisted despite efforts to identify contributing factors. Despite controlling for income, insurance, age, gender, BMI and language spoken, Black patients were 37% less likely to receive TKA than White, non-Hispanic patients from 2015 to 2019 [3]. In a separate study, time from orthopedic consult to surgery did not explain the disparity in utilization or access to surgery [9]. Socioeconomic status (SES) and race are historically intertwined. Despite efforts to control for the effect of SES on TKA utilization, proxies for SES are known to be imperfect and there is likely residual confounding or intermediary effects.

During the early months of the COVID pandemic, utilization rates for elective total joint arthroplasty (TJA) fell abruptly. Compared to white patients, rates for non-White patients fell more drastically, took longer to recover and never regained their 2019 utilization levels. Averaging TKA utilization changes from April to December 2020 and comparing against prior years, utilization of TKA for Black patients fell by 36%, compared to 27% for white patients (p < 0.001) [10].

### 3.2 Knee Replacement Outcomes

Key TKA outcomes identified included pain and function, patient satisfaction, and complications of surgery, which includes rates of revision surgery, readmissions, emergency department (ED) visits, morbidity and mortality. Length of stay and non-home discharges are other important metrics, the latter of which is more likely to impact patient satisfaction or other patient reported outcome measures (PROMs).

#### 3.2.1 Differing pain and functional outcomes after Knee Replacement Surgery

Racial differences in PROMs have been investigated mostly in single or smaller multi-center studies. One large study looked at a 3-center Veterans Health Administration study (TKA N = 587, 7.7% Black, total hip arthroplasty (THA) N = 271, 9.2% Black). Black patients were found to have less improvement in Knee Injury and Osteoarthritis Outcome Scores for Joint Replacement (KOOS JR) after TKA (19.8 vs. 16.6, p-value = 0.05) [11].

A possible explanation of the poorer Black post-operative physical function (PF) is the disparity in use of physical therapy (PT). Using PearlDiver data from 2014 to 2016, Ratnasamy et al. found that Black patients (N = 1250) are significantly less likely to utilize PT in the 90 days after TKA when adjust for age, sex, and Elixhauer comorbidity index [12]. Another large study of 10,325 individuals using the Women's Health Initiative data set looked at TKA in women with Medicare and found that Black women had lower PF scores based on the Research and Development Corporation 36-Item Health Survey than non-Hispanic White women both preoperatively (mean difference −5.8, 95% CI [−8.0, −3.6]) and 1-year postoperatively (7.8 points lower, 95% CI [−10.8, −4.9]) and at all time points up to 10 years [13].

Other authors have attributed poorer post-operative PF (measured by KOOS) for Black patients to poorer pre-operative PF status. In a single-center study of 5284 patients, Black patients undergoing TKA had lower preoperative PF relative to White patients 33.5 vs 45.1, p-value<0.001), but at 6 weeks and 1 year post TKA, there was no significant difference in PR, demonstrating greater postoperative PF gains for Black patients [14].

Rahman et al. found that there was no significant difference in PROMs after TKA based on KOOS JR between Social Vulnerability Index and Area Deprivation Index quartiles, which, given the racial disparities in distribution of Social Vulnerability Index and ADI, further suggests no significant racial disparities in PROMs. This study also had a large sample size (N = 19,321 TKA cases) and utilized the Michigan Arthroplasty Registry Collaborative Quality Initiative database [15].

#### 3.2.2 Differing operative complications

There is a substantial body of literature, beyond patient reported outcomes, investigating disparities in complications after TKA, post-operative mortality, readmission rates, ED visits, and revision rates.

Operative time was found to be significantly longer for patients of Black race undergoing THA, but the data was mixed in TKA with some research demonstrating no difference with other studies supporting longer operative time for Black patients, potentially due to one study utilizing a nationally representative survey while the other was based in a single institution [16,17].

Effects of race and ethnicity on readmission, medical and surgical complications after TKA were examined using the American College of Surgeons National Surgical Quality Improvement Program (ACS-NSQIP), for over 600 hospitals and 262,954 patients with self–reported race and/or ethnicity who underwent primary, elective TKA between 2011 and 2017 [16]. The authors found that Black patients had significantly higher rates of readmission (1.14, 95% CI [1.05, 1.24]) and surgical complications (OR 1.23, 95% CI [1.11–1.37]) than White patients, though there was no difference in 30-day mortality [16].

Looking at length of hospital stay, 90-day readmission and post-operative mortality, Munir et al. found Medicare Black patients undergoing TKA or one of 4 other surgical procedures during 2013–2017 (where TKA patients were 75% of the sample) had worse composite quality of metric scores comprised of LOS, 90-day readmission, post-operative complication and 90-day mortality (OR 0.78, 95% CI [0.76,0.79]) [18].

We identified one prospective single-center study that implemented an Enhanced Preoperative Education Pathway care path for high-risk patients that included Black patients, with the goal or reducing disparities in ED visits (and other post-operative outcomes). The study did demonstrate reduced ED visits for Black patients, but it was not randomized, and it did not lead to change in 30-day readmission rates, length of stay or non-home discharges [19].

Early revision surgery may be a complication or failure of joint replacement. In a review of revision TKAs from the American Joint Replacement Registry from 2012 to 2020, Black patients were more likely to undergo revision surgery compared to White patients (HR = 1.26 95% CI [1.13, 1.40]). Using National Inpatient Sample data from 2006 to 2014, Black patients were found to be 13% more likely to undergo revision TKA compared with White patients within 2 years of surgery, with these disparities worsening overtime [20]. A study using NSQIP data from 2008 to 2020 found that Black patients had significantly lower rates of revision TKA when compared with White patients who underwent outpatient or short-stay TJA [21]. Some studies did not find race-based differences for post-operative mortality or 30-day readmission rates, but a recent, large meta-analysis of TJA utilization and complications did find significant difference in 30-day complications (TKA complications OR1.20, 95% CI [1.10, 1.31]) [8].

Studies have evaluated discharge location and found that Black patients were more likely to discharged to non-home settings than White patients [14,[22], [23], [24], [25], [26], [27]]. A recent meta-analysis summarized the effect of TKA studies finding that Black patients had an adjusted OR 1.65 (95% CI [1.38, 1.96]) of non-home discharge over White patients [8]; and for combined THA and TKA, Black patients were more likely (OR 1.91, 95% CI [1.62, 2.25]) to be discharged to institutional rehabilitation or a skilled nursing facility [8]. Only one single center study from Michigan found no significant racial differences in non-home discharges [15].

In contrast to other data presented in this review, one single-center study at a minority-majority safety net hospital in the Bronx, New York (n = 3,093, 47.9% Hispanic, 38.3% Black), Black patients had significantly shorter length of hospital stay and decreased likelihood of non-home discharge when compared to patients of White race [28].

In a retrospective single institution study, Distressed Communities Index was found to be associated with increased 90-day readmissions and increased post-operative prescriptions for TKA. In another study to examine the impact of neighborhood characteristics (ADI and other neighborhood characteristics) on hospital readmission rates and ER visits after TJA, these neighborhood characteristics were more likely to negatively impact readmission and ER outcomes for Black patients than White patients [29]. We found only one large multicenter retrospective study that did not find race to be independently associated with readmissions [30].

#### 3.2.3 Patient perceptions of benefits of surgery

In a systematic review of patient satisfaction in TKA [31], two papers identified Black race as an independent risk factor for poorer satisfaction after TKA. Burke et al. likewise investigated racial disparities in satisfaction regarding joint replacement surgery. Using Press Ganey scores from THA or TKA hospitalizations at a tertiary care center from 2010 to 2012, they found that patients of Black race were significantly less likely to be satisfied with their hospital experience and nursing care [32].

### 3.3 Patient Characteristics

We identified 28 articles that evaluated the impact of patient characteristics on TJA decision-making. We discovered the following patient characteristics during our review process: patient PF status, frailty, underlying severity of arthritis, pain catastrophizing, medical comorbidities, age, sex/gender and education.

Rates of diabetes, hypertension, anemia, smoking, heart failure and overall comorbidity burden were noted to be higher in the Black patient population [16,22,33]. Even when controlling for comorbidities, disparities persisted in many studies, including in TJA utilization [34] and postoperative complications and readmissions [16]. However, other studies found that when controlling for comorbidities, race was not an independent risk factor for unfavorable outcomes, including 30-day postoperative complications, location of surgery [33], and likelihood to be referred to a specialist [35]. Wu et al. found that comorbidities played an important role in contributing to postoperative complications and proposed addressing the comorbidity burden that disproportionately affects Black patients as a means to reduce inequities in TJA [33]. Likewise, Mickle et al. found that when controlling for environmental and behavioral factors, differences in pain and PF decreased between non-Hispanic White and non-Hispanic Black individuals [36].

Obesity is a relative risk factor for elective TKA. Obesity is also known to be more prevalent among Black adults and Hispanic adults [37]. To explore whether obesity affects the observed racial and ethnic disparities in TKA and THA utilization, a retrospect review of data from the American College of Surgeons National Surgical Quality Improvement Program (ACS-NSQIP) database (2015–2019) and the National Health and Nutrition Examination Survey (2011–2018) was conducted [38]. The analysis supported the conclusion that greater prevalence of obesity in above groups may adversely affect eligibility for TKA and THA.

Multiple articles noted disparities in severity of baseline OA between Black patients and White patients [39,40]. Suleiman et al. found that Black patients were more likely to present with severe OA but reported levels of joint dysfunction similar to those of White patients, underscoring important differences in perceptions of arthritis [41]. It is possible that disparities at multiple points along the TKA referral pathway lead to Black patients with OA receiving TKA later in the disease course than White patients.

### 3.4 Cultural Characteristics

We classified articles that addressed the role of cultural characteristics in joint arthroplasty into two subtopics: degree of trust in the healthcare system and value of complementary and alternative medicine. Both topics play an important role in a patient's decision to undergo joint replacement surgery, but disparities persist that influence the decision-making process.

#### 3.4.1 Trust in healthcare system

Of the 6 articles that addressed racial differences in degree of trust in the healthcare system, the majority of studies found that Black patients have less trust and poorer perception of TJA than White patients based on patient interview and surveys. Only one article failed to identify a significant difference in trust in the healthcare system [42]. Mingo et al. and Chang et al. found that Black participants were more likely to report lack of trust in the healthcare system based on structured questionnaires [43] and, conversely, that White American men tended to not place importance on issues of trust in physicians when prompted to raise concerns about joint replacement surgery in focus groups [44].

#### 3.4.2 Value of complementary and alternative medicine

We found differences in preferences between Black study participants and White participants in the value of complementary and alternative medicine. Katz et al. found Caucasian participants in their study were significantly less likely to use complementary and alternative medicines compared to Black participants (OR 0.5, 95% CI 0.3–0.8) [45].

In particular, Black participants in multiple studies were more likely to use prayer for treatment of arthritis and surgery decision-making [22,[46], [47], [48], [49]]. In one notable study that specifically assessed the role of prayer on consideration of joint replacement surgery, Ang et al. found that respondents who noted the helpfulness of prayer in management of arthritis were less likely to consider arthroplasty (OR 0.64, 95% CI 0.43–0.94), though this difference did not vary across ethnic backgrounds [49].

Based on data from the MarketScan database from 2012 to 2019, Black patients were less likely to use prescription pharmacologic meds (duloxetine, oral or intramuscular glucocorticoids or opioids), more likely to use acetaminophen and topical analgesics, less likely to receive intra-articular injections (particularly for hyaluronate) and less likely to receive PT, transcutaneous stimulator, acupuncture or chiropractic care [50].

### 3.5 Structural Barriers

Barrier such as healthcare policies, distribution of resources, and patient-provider relationships can affect the likelihood of TKA for a particular patient.

#### 3.5.1 Access to high volume joint replacement hospitals and surgeons

Distribution of resources can affect access and patient outcomes. Losina et al. showed that in 2007, non-white Medicare patients undergoing TKA were twice as likely to receive care at low volume hospitals (LVH) than white patients and twice as likely (16% of the time) to drive past a high volume hospital to receive that care [51]. From a 100% national sample of Medicare patients undergoing TKA, in densely populated urban areas (≥50,000 residents), African-American (OR = 1.55) and other minority races (OR = 2.27) are more likely to have their surgery at LVH than Caucasian patients undergoing TKA [52]. Both of these studies showed that reduced access to higher quality hospitals was due to factors beyond distance.

LVH were more likely to be rural, have smaller bed size, and patients undergoing THA/TKA at LVH were more likely to be admitted for non-elective indications, have Medicaid insurance and higher post-surgical complication rates and longer length of stay. Given these differences in insurance, the role of income and SES are difficult to extricate from the effects on TKA utilization in the abovementioned study. Likewise, Orringer et al. found that White, Medicare and commercially insured patients would travel farther than black patients to receive TKA [53].

#### 3.5.2 Likelihood to Be recommended surgery

Through a survey of 198 racially diverse patients (27% Black), TKA was discussed more frequently with White patients and White patients were more frequently to consider TKA than Black patients [42]. Black patients were less likely to have friends or relatives to have undergone TKA. While Black patients had less trust in the health care system (than White patients), there were similar levels of trust in treating physicians.

In a prospective study of 120 Black and 337 White Veterans with moderate to severe knee OA (WOMAC >39) scheduled for orthopedic consultation, Black patients were offered surgery at half the rate (adjusted OR 0.46) of White patients [54]. However, after adjusting for patient stated preference, where 68% of White but only 49% of Black patients were definitely willing to consider TJA, the adjusted OR of receiving a recommendation for TJA was attenuated to 0.78 and no longer significant.

## 4 Discussion

Our scoping review covers disparities throughout TKA decision-making. Across the studies identified in this review, Black patients have more severe pain, poorer function and higher rates of medical comorbidities such as hypertension diabetes, obesity, heart failure, and tobacco use at time of surgical evaluation. Black patients are less likely to expect positive outcomes after TJA, are more likely to prefer alternative treatments for osteoarthritis, and have a lower degree of trust in the healthcare system. The results identified in this study do not represent the entire population of Black patients with knee OA considering TKA but highlight the current evidence for how disparities affect the TKA evaluation process.

Adverse events after surgery are more frequent for Black patients compared to White patients. This includes medical and surgical complications, non-home discharges, likelihood to require revision surgery, length of stay, and operative time.

Well intended policy payment models have resulted in unintended consequences with increased rates of readmissions, ED visits, non-home discharges, and medical and surgical complications (summary of policy reviews in supplemental section). These complications all result in higher episode-of-care costs for Black patients.

Targeted interventions have had beneficial outcomes. One example is the enhanced preoperative education care pathway, which was been shown to cause a reduction in ED utilization for Black patients without increase in readmissions [19]. Liu et al. investigated the use of evidence based perioperative practice in TKA and THA, and found that Black patients had lower rates of evidence based perioperative practice utilization compared with White patients [55]. Clearly, given the disparities at all steps in the decision-making tree, interventions will need to be targeted at multiple points in the process. Similar education campaigns could theoretically improve awareness of the benefits of TJA.

Our study has several limitations. The data summaries in this review are limited to qualitative summaries given the high heterogenicity, which made performing meta-analyses impractical. The lack of consistency in outcome measures across studies introduces the possibility that measurement bias contributes to some of the differences in outcomes and access to care that are noted in this review. Future studies should seek to establish consensus regarding outcome measures that accurately identify disparities in TKA utilization. Additionally, many of the articles analyzed are retrospective studies using similar or, in some cases the same databases, sampled at different times. Furthermore, the studies present in this review were published between 1989 and 2024, a period during which surgical techniques, perioperative management and the United States healthcare system drastically changed, which potentially limits the relevancy of this study. However, the purpose of this study is not to identify temporal trends or shifting inequities in TKA utilization, but rather to present a complete review of the TKA decision-making process wherein disparities can emerge.

All studies included in this review are taken from data generated within the United States, which limits the generalizability of these results to other countries. Further studies that address the role of healthcare disparities in TKA unique to the demographic composition, healthcare systems and historical inequities present in other national contexts are warranted. We identified a dearth of studies that address provider-level factors, which, as can be seen in our model, contribute to TKA utilization. Future research should address the influence of institutional effects, surgeon experience and provider characteristics on TKA disparities. Finally, due to changes in team structure that occurred in response to the COVID-19 pandemic and trainees graduating, there were discrepancies in the reviews by the two teams. This was addressed by the repeat review of the identified references.

## 5 Conclusion

There are racial disparities between Black and White patients at multiple steps in the decision to offer joint replacement surgery. This includes the prevalence of baseline comorbidities, degree of trust in the healthcare system, severity of radiographic osteoarthritis at presentation, functional outcomes, post-operative medical and surgical complications, increased likelihood to require revision surgery, rates of non-home discharges, ED visits, and readmissions. Modifiable factors (based on misinformation, racial bias or structural barriers) ought to be targets for intervention. Non-mutable factors or factors such as patient preference (e.g. for alternative care) or risk aversion (due to poorer observed outcomes) are not optimal targets. We propose a new model for evaluating the steps that lead to joint replacement and joint replacement disparities for which future interventions could be targeted.

## Author contributions

All authors confirm that they have participated in the conception and design of the study, analysis and interpretation of data, drafting the article or revising it critically for important intellectual content, and final approval of the version to be submitted. Samuel Good takes responsibility for the integrity of the work as a whole.

## Declaration of Generative AI

Generative AI and AI-assisted technologies were NOT used in the preparation of this work.

## Funding source

Arthritis Foundation: Diversity, Equity, and Inclusion (DEI) Award.

## Conflict of interest

SDG: none.

MK: none.

CB: none.

DPF: none.

CK: none.

AP: consultant for Stryker, Smith and Nephew, Naviswiss, AccuPredict, Osgenic, Revelai.

MD: none.

SG: none.

JDF: none.
