# Supplementary material for: Racial Differences in Utilization of and Factors Contributing to the Use of Knee Replacement Surgery as a Treatment for Severe Knee Osteoarthritis: A Scoping Review
Source: Osteoarthr Cartil Open. 2026 Jun 22;8(3):100844. doi: 10.1016/j.ocarto.2026.100844 (PMC13330662; doi:10.1016/j.ocarto.2026.100844)
Supplement: Multimedia component 1 [file mmc1.docx]

**Supplemental Material**

Potential Policy Impact on Disparities in Care

Though Medicare covers TJA and is frequently cited as “leveling the playing field” by reducing financial barriers, changes in policy can have differential impacts by race or ethnicity. In the Health and Retirement Study the lower Black American utilization rates for THR and TKR was only significant for Blacks > 65 years of age^1^ and not for younger (age < 65 years) Black patients. However, this may have been due to smaller sample size of young Black patients with arthritis (n = 1200.) Other authors have postulated that access to Medicare insurance might reduce disparities in TJA utilization, but that was not seen in this study.

Given the large annual Medicare expenditure on TJA, there have been several fiscal policy interventions designed to reduce (potentially wasteful) expenditures, improve patient outcomes, or improve equitable access to care. The Inpatient Prospective Payment System and Diagnostic Resource Groups (bundled hospital payments) introduced in 1983, were designed to promote efficient use of hospital resources. The Short Stay Transfer Policy (with penalties for “early” discharge to post-acute care) implemented in 1998, was designed to improve efficient use of post-acute care resources.

The Patient Protection and Affordable Care Act (PPACA) of 2010 was implemented with the goals of improving access to care, optimizing quality, while reduction in costs. A retrospective review of NIS dataset showed a marked increase favoring use of Urban teaching hospitals (increasing from 38% to 58%) with fall in use (48% to 31%) of Urban non-teaching hospitals for delivery of TKR.^2^ There was slight increase in the age-adjusted proportion of Black patients receiving TKR and patients from lower incomes without clear findings that access was improved.

Introduced as Medicare part C with the Balanced Budget Act of 1997, and rebranded as Medicare Advantage (MA) in 2003, participation in the program increased from 7.9% in 2004 to 34.4% 2020.^3^ Using a nationally representative database (Premier Healthcare Database), Wang and colleagues examined patients undergoing TKR or THR between 2015 and 2020. A higher proportion of MA patients undergoing TJR were black (8.3% vs. 4.6%) than patients covered by traditional Medicare. MA hospitals were more likely urban, a little larger but with lower surgeon and hospital TJR volumes. MA patients had more comorbidities (not explored by race) and more frequent adverse outcomes (including periprosthetic joint infection).

The Comprehensive Care for Joint Replacement (CJR) model, implemented April 2016 in 34 (11% of) Metropolitan Statistical Areas (MSA), (expanded October 2021 to another 33 MSA), bundled Medicare’s inpatient and post-surgical care in the 90 days after discharge with the intent to improve quality of care and reduce Medicare expenditures per procedure.^4^ However, the CJR did not include risk adjustment for patients’ preexisting social or medical complexity and therefore could restrict access to care for more vulnerable patient groups. Not all Metropolitan regions participated in the payment model, thus creating conditions to evaluate differential impact of the policy. In the large 2013-2017 national Medicare claims sample, no change TJR utilization rates between treatment and control regions (+0.04 increase per 1000 patient-years, p = NS) for White patients. There was higher TJR utilization for Hispanic patients (+1.6 per 1000 patient-years) in CJR regions and lower TJR utilization (-0.64 per 1000 patient-years) for Black patients in CJR regions (after multivariate analyses).

Using the same dataset, Thirukumaran and colleagues further looked at TKR vs. THR and impact of dual-eligible status (Medicaid-Medicare recipients). There was larger negative impact on utilization for Black patients undergoing TKR, compounded further by dual eligibility status than for THR.^5^

However, using a 20% sample of fee-for-service Medicare inpatient claims for April 1, through December 31, 2015 and April 1, through December 31, 2016, difference-in-difference models showed that despite large differences in hospital size and racial mix of patients who were affected by CJR, there was no significant differential impact on utilization by black race or dual eligibility status.^6^

Removal of TKR (but not THR) from the Medicare Inpatient Only (IPO) designation status in 2018 resulted in 11% of Medicare covered TKR (in New York state) being performed in outpatient setting.^7^ In carefully selected patients, outpatient TKR does not have poorer surgical outcomes. Hospitals offering outpatient TKR are larger, and less likely to have disproportionate share (DSH) hospital payments. Black, non-Hispanic patients are less likely to undergo outpatient TKR compared to other race/ethnicities.^7^

Citations

1. Dunlop DD, Manheim LM, Song J, Sohn MW, Feinglass JM, Chang HJ, et al. Age and racial/ethnic disparities in arthritis-related hip and knee surgeries. *Medical care*. 2008;46(2):200-208. doi:10.1097/MLR.0b013e31815cecd8

2. Gwam CU, Mohamed NS, Etcheson JI, Davila Castrodad IM, Mistry JB, Recai TM, et al. Changes in Total Knee Arthroplasty Utilization since the Implementation of ACA: An Analysis of Patient-Hospital Demographics, Costs, and Charges. *Journal of Knee Surgery*. 2020;33(7):636-645. doi:10.1055/s-0039-1683926

3. Wang J.C., Piple A.S., Chen X.T., Bedard N.A., Callaghan J.J., Berry D.J., et al. The Rise of Medicare Advantage: Effects on Total Joint Arthroplasty Patient Care and Research. *Journal of Bone and Joint Surgery*. 2022;104(24):2145-2152. doi:10.2106/JBJS.22.00254

4. Kim H, Meath THA, Quinones AR, McConnell KJ, Ibrahim SA. Association of Medicare Mandatory Bundled Payment Program With the Receipt of Elective Hip and Knee Replacement in White, Black, and Hispanic Beneficiaries. *JAMA Network Open*. 2021;4(3):e211772. doi:10.1001/jamanetworkopen.2021.1772

5. Thirukumaran CP, Kim Y, Cai X, Ricciardi BF, Li Y, Fiscella KA, et al. Association of the Comprehensive Care for Joint Replacement Model With Disparities in the Use of Total Hip and Total Knee Replacement. *JAMA Network Open*. 2021;4(5):e2111858. doi:10.1001/jamanetworkopen.2021.11858

6. Humbyrd C.J., Wu S.S., Trujillo A.J., Socal M.P., Anderson G.F. Patient Selection After Mandatory Bundled Payments for Hip and Knee Replacement: Limited Evidence of Lemon-Dropping or Cherry-Picking. *Journal of Bone and Joint Surgery*. 2020;102(4):325-331. doi:10.2106/JBJS.19.00756

7. Schloemann DT, Sajda T, Ricciardi BF, Thirukumaran CP. Association of Total Knee Replacement Removal From the Inpatient-Only List With Outpatient Surgery Utilization and Outcomes in Medicare Patients. *JAMA network open*. 2023;6(6):e2316769. doi:10.1001/jamanetworkopen.2023.16769
